# Resolution of stuttering during ketamine treatment: a case report

**DOI:** 10.1186/s13256-023-04158-8

**Published:** 2023-10-10

**Authors:** Dan Bolton, Tegest Hailu, Christina A. Porucznik

**Affiliations:** 1 Health Care Research Center, Washington State Office of Financial Management, Olympia, WA USA; 2 Providence SoundHomeCare and Hospice, Lacey, WA USA; 3 Division of Public Health, Department of Family and Preventive Medicine, University of Utah, Salt Lake City, UT USA

**Keywords:** Stuttering, Ketamine, Depression, Case report

## Abstract

**Background:**

Stuttering may include repetition of words in whole or part, difficulty saying words, and elongated pauses in speech. Approximately 5% of children stutter for a period lasting 6 months or more. Most of those children stop stuttering as they approach adulthood, but the condition persists in approximately 1% of adults. The cause of stuttering is unknown. Adults who stutter face substantial burdens in many aspects of their lives. Stutterers may choose not to pursue meaningful employment opportunities, may not be hired for positions they seek, or may be denied promotions or positive performance evaluations. Stuttering can cause physical tension from fear of speaking. Social challenges arise when a person who stutters is seen as less capable or of lower intelligence than fluent speakers. Stuttering causes emotional difficulties through the frustration and embarrassment that disfluent speakers feel. Stutterers may experience a general loss of self-esteem and personal satisfaction in life. Speech therapy is the primary intervention for stuttering. Medications have been investigated as treatments for stuttering, but no medication has been identified that has widespread effectiveness.

**Case presentation:**

A 60-year-old white non-Hispanic woman who had been a near lifelong stutterer was prescribed ketamine for an unrelated condition and experienced an almost immediate resolution of her stuttering.

**Conclusions:**

Many possible pharmacological treatments for stuttering have been studied. Some medications appear to be effective in some patients; some appear to be more generally effective but have negative side effects. No reporting in relevant literature has addressed a possible role for ketamine in stuttering treatment. On the basis of this case report, research on the effect of ketamine on stuttering would be useful. Any effective treatment for stuttering would have a significant positive effect on quality of life for persons who stutter.

## Background

A meta-review of stuttering research notes “[t]here is no consensus on the pathophysiology of stuttering” [1]. The authors list possible mechanisms including differences in brain anatomy and function, variations in regulation of dopamine, and genetic causes, but finally state that “clear mechanisms of actions have yet to be identified;” another study likewise notes that “the etiology of the condition is unclear” [2].

Childhood stuttering often resolves with time, but is less likely to do so when it continues after a child reaches 8 years [2]. Researchers estimate that less than 1% of the population are adults who continue to stutter after childhood [1]. Adults who stutter display anxiety as well as “lower quality of life, occupational and educational burdens, and barriers to receiving high-quality health care”; these challenges can include serious difficulties in educational and employment settings [1, 2].

Researchers identify three categories of stuttering: developmental, neurogenic, and psychogenic. Most people who stutter have developmental stuttering. Neurogenic stuttering is generally associated with some form of brain damage, such as a stroke. Psychogenic stuttering “usually occurs in adults with a history of psychiatric problems following a psychological event or emotional trauma” [2].

Many drugs have been suggested as treatment for stuttering. Sometimes treatments are proposed, other times a drug prescribed for a different condition appears to influence stuttering. Drugs that have been studied for possible effects on stuttering have included anticonvulsants [3], antidepressants [4], antipsychotics [5], beta blockers [6], botulinum toxin [7], selective serotonin reuptake inhibitors (SSRIs) [8], levodopa [9], and dopamine receptor blockers [10].

Research continues, but no universal pharmacological treatment has been identified. Some drugs seem to benefit some stutterers but not others. At least one medication with a possible impact was also found to have serious negative side effects. No published report has suggested ketamine as a treatment for stuttering.

The Food and Drug Administration (FDA) approved ketamine for use as an anesthetic in human patients in 1970. It is a class III controlled substance [11, 12]. When given at doses lower than those that induce anesthesia, ketamine has an antidepressant effect and has been prescribed off-label as a treatment for depression.

## Case presentation

The patient was an adult white non-Hispanic female stutterer who stopped stuttering after being prescribed ketamine for depression. The patient stuttered through most of her life, beginning in childhood. She was a developmental stutterer and did not experience seizures or any other conditions associated with neurogenic or psychogenic stuttering. She began speech therapy in elementary school but did not receive therapy continuously. She participated in further therapy while attending university in her 20s, then later in her 20s and 30s after completing her studies, and finally in her 40s and 50s. Her last treatment was in her 50s, approximately 2 years before she was enrolled in hospice care.^1^ The patient at times experienced improvement in her speech as a result of therapy, but the improvement was not permanent. The patient’s course of treatment for stuttering did not include physical examinations. Her father also stuttered during childhood and adolescence; his stuttering resolved spontaneously when he was in his 20s.

The patient suffered from chronic lung disease and stage 4 chronic kidney disease and was eventually enrolled in hospice care at time T = 0. Hospice providers prescribed hydromorphone and methadone for pain relief, and lorazepam for anxiety. The patient’s other medications were discontinued when she began hospice care. No change in the patient’s stuttering was apparent while she took the first medications prescribed through hospice. The patient began ketamine treatment at time T = 5 months after entering hospice. Physical examinations during hospice care were conducted by a hospice nurse in consultation with the treating physician. The examinations focused on the patient’s vital signs, the efficacy of pain relief, diet, and the patient’s overall comfort.

While in hospice care the patient was diagnosed with depression. Her treating physician prescribed a low dosage of ketamine (0.25 ml, 10 mg/1 ml, twice daily) for depression. By contrast, dosages of ketamine for treatment-resistant depression may range between 0.5 mg and 1 mg per kg of body weight.

The patient was also prescribed haloperidol as part of hospice care, but she had earlier had a negative experience with haloperidol and took it only once or twice, discontinuing the medication before she began treatment with ketamine.

Within 24 hours of beginning ketamine treatment, the patient experienced a resolution of her stuttering. She was in frequent contact with family and close friends. They had been familiar with her stuttering for many years, in some cases from its inception. They noticed and commented on the change almost immediately. There was some limited breakthrough stuttering in the form of repetitions, but there were no blocks in the patient’s speech. This outcome continued until the patient’s death, approximately 1 month after she began ketamine treatment and T = 6 months after she entered hospice care. Once she started the ketamine prescription, the patient took it continuously until her death. The timeline of events made it impractical to discontinue and then reintroduce the medication. The resolution of her stuttering improved her spirits. It may not be possible to say whether the patient’s depression was alleviated separately by the ketamine treatment, her newly fluent speech, or a combination of the two.

## Discussion and conclusions

Drug categories that have been considered as treatments for stuttering were previously identified in the background section. A few of those medications are relevant in this case. More than 20 publications discuss haloperidol as a treatment for stuttering. There appears to be some evidence for its effectiveness, but that effect is countered by side effects that make it unsuitable in this context. As noted, the patient here was briefly treated with haloperidol. The medication had no observable effect on her stuttering.

Two medications that appear in the literature—gabapentin and baclofen—merit specific mention because the patient had taken them before hospice enrollment. The patient discontinued both medications no later than her enrollment in hospice care at T = 0, 5 months before she began ketamine treatment.

Gabapentin is cited as both a cause of stuttering [13] and a treatment for it [14]. The patient was prescribed gabapentin for neuropathic pain. Her treatment with gabapentin had no apparent effect on her stuttering. She continued speech therapy during this time with no mention of that drug or any effect in relation to stuttering.

A 2017 study described a patient who was treated with baclofen for alcohol dependence who also stopped stuttering while taking the medication [15]. Here, the patient was prescribed baclofen for muscle pain and tightness, with no apparent effect on her stuttering.

The patient was a lifelong stutterer whose speech difficulties took a significant toll on her personal and professional life. She several times sought help through speech therapy. The therapy was sometimes helpful, but she finally discontinued it in her late 50s because she was not seeing progress in her speech.

The impact of the ketamine prescription’s unexpected effect was substantial. The patient could consistently speak freely for the first time since early childhood. This effect itself gave relief from depression as she communicated with ease. For this patient, the ketamine prescription was life-changing.

A review of the literature on attempts to treat stuttering with medication revealed no mention of ketamine.

In this case the treating physician prescribed a low dose to help with pain relief and end-of-life depression issues. The physician sometimes titrates the dose up as tolerated but that did not happen here. As noted, ketamine is also used for treatment-resistant depression. The potential link between ketamine as a treatment for depression and a possible effect on stuttering suggests it would be wise to screen patients who stutter for depression as part of their care.

It is challenging to assess how the ketamine treatment might have affected the patient’s stuttering. As noted, although researchers have identified possible causes of stuttering, the precise pathogenesis is not understood. In turn, that lack of clarity complicates efforts to specify how a drug might affect stuttering. Ketamine’s effect on the patient’s stuttering may relate to its effect on the dopamine system. Recent research cites growing evidence suggesting that “dopamine antagonist medications are effective in reducing the severity of stuttering symptoms” [16]. Other research on ketamine and neuropsychiatric disorders discusses ketamine as a dopamine transporter antagonist in the context of its effect on dopaminergic function, while noting that anesthetic doses may act differently from subanesthetic doses [17]. Further research into ketamine’s effects would likely shed light on its possible effect on stuttering.

This case thus extends the existing literature on pharmacological treatment of stuttering by identifying a medication that apparently affected a patient’s stuttering. There has been no documentation of ketamine affecting stuttering. Further research on ketamine as a possible treatment for stuttering would be valuable and could lead to improved outcomes for stutterers.

Stuttering burdens many aspects of the lives of those with the condition. Many medication-based approaches to treatment have been explored with mixed results, but no solution that works for all stutterers has been identified. The patient in this case report experienced near-lifelong stuttering that was not consistently responsive to therapeutic methods. Medication prescribed for a different condition had the unexpected result of resolving her stuttering and enabling her for the first time in decades to communicate through speech without impediment. It is possible that treatment with the same medication could benefit other stutterers. Researchers who study pharmacological treatment for stuttering should investigate further to determine whether others could so benefit.

## Data Availability

Data sharing is not applicable to this article as no datasets were generated or analyzed during the current study.

## Abbreviation

**FDA**: The United States Food and Drug Administration

## Footnotes

1 It is not possible to report on the severity of the patient’s stuttering. The authors interviewed one therapist who had assessed severity. However, the therapist refused to provide the results of that evaluation, citing the Code of Ethics of the American Speech-Language-Hearing Association. The request was refused despite consent from the patient’s legally authorized representative.

## References

[1] Perez HR, Stoeckle JH (2016). Stuttering: clinical and research update. Can Fam Phys.

[2] Prasse JE, Kikano GE (2008). Stuttering: an overview. Am Fam Phys.

[3] Baratz R, Mesulam M-M (1981). Adult-onset stuttering treated with anticonvulsants. Arch Neurol.

[4] Costa D (1992). Antidepressants and the treatment of stuttering. Am J Psychiatry.

[5] Charoensook J, Maguire GA (2017). A case series on the effectiveness of lurasidone in patients with stuttering. Ann Clin Psychiatry.

[6] Burris JF, Riggs MC, Brinkley RR (1990). Betaxolol and stuttering. Lancet (London, England)..

[7] Brin MF, Stewart C, Blitzer A, Diamond B (1994). Laryngeal botulinum toxin injections for disabling stuttering in adults. Neurology.

[8] Busan P, Battaglini PP, Borelli M, Evaristo P, Monti F, Pelamatti G (2009). Investigating the efficacy of paroxetine in developmental stuttering. Clin Neuropharmacol.

[9] Anderson JM, Hughes JD, Rothi LJG, Crucian GP, Heilman KM (1999). Developmental stuttering and Parkinson’s disease: the effects of levodopa treatment. J Neurol Neurosurg Psychiatry.

[10] Rothenberger A, Johannsen HS, Schulze H, Amorosa H, Rommel D (1994). Use of tiapride on stuttering in children and adolescents. Perceptual Motor Skills [Internet]..

[11] DEA Diversion Control Division. Controlled Substances—Alphabetical Order—DEA CSA SUBSTANCE NUMBER SCH NARC OTHER NAMES [Internet]. 2016. Available from: https://www.deadiversion.usdoj.gov/schedules/orangebook/c_cs_alpha.pdf. Accessed 2 May 2023.

[12] U.S. Code § 812—Schedules of controlled substances [Internet]. LII/Legal Information Institute. 2012. Available from: https://www.law.cornell.edu/uscode/text/21/812. Accessed 2 May 2023.

[13] Nissani M, Sanchez EA (1997). Stuttering caused by gabapentin. Ann Internal Med..

[14] Maguire GA, Bird AA (2012). Gabapentin for treating acquired neurogenic stuttering. Ann Clin Psychiatry.

[15] Beraha E, Bodewits P, van den Brink W, Wiers R. Speaking fluently with baclofen? BMJ Case Reports. 2017; bcr-2016-21871. 10.1136/bcr-2016-218714 PMC5534838 28495786

[16] Maguire GA, Nguyen DL, Simonson KC, Kurz TL. The pharmacologic treatment of stuttering and its neuropharmacologic basis. Front Neurosci. 2020;14. 10.3389/fnins.2020.00158 PMC7118465 32292321

[17] Kokkinou M, Ashok AH, Howes OD (2017). The effects of ketamine on dopaminergic function: meta-analysis and review of the implications for neuropsychiatric disorders. Mol Psychiatry.

